# Supplementary material for: Identification of HIV-1 Envelope Mutations that Enhance Entry Using Macaque CD4 and CCR5
Source: Viruses. 2020 Feb 21;12(2):241. doi: 10.3390/v12020241 (PMC7077290; doi:10.3390/v12020241)
Supplement: Supplementary file 1 [file viruses-12-00241-s001.pdf]

Figure S1

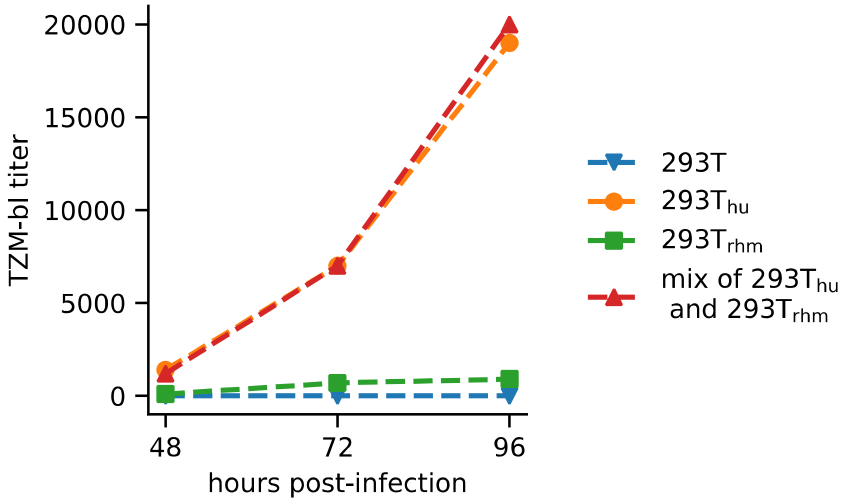

Figure S1. Wild-type BF520 infection of 293T cells engineered to express either macaque or human CD4/CCR5 receptors. TZM-bl titer of supernatant harvested from cells infected with viruses bearing the BF520 Env (MOI of 0.01). The cells that were infected are shown to the right. "Mix of 293T$_{hu}$ and 293T$_{rhm}$" refers to a 3:1 mixture of these cells, respectively. Each data point reports the titer of one viral supernatant sample assayed in triplicate on TZM-bl cells.

Figure S2

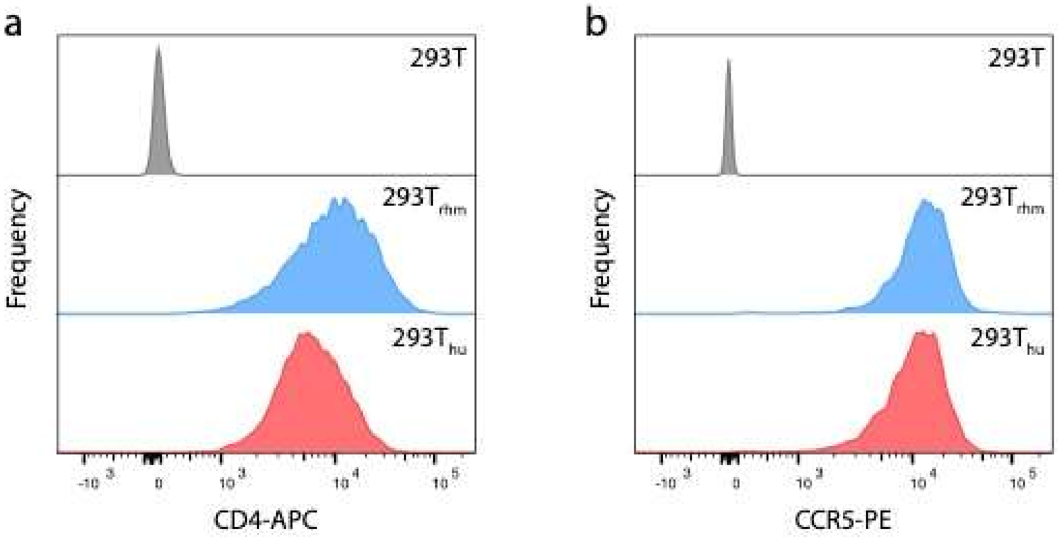

Figure S2. Expression of CD4 and CCR5 on $293T_{rhm}$ and $293T_{hu}$ cells. Expression of CD4 (a) and CCR5 (b) on un-transduced 293Ts, $293T_{rhm}$, and $293T_{hu}$ cells. For each density plot, receptor expression on 10,000 cells taken from one biological sample was measured. Data is representative of multiple independent experiments.

Supplementary Table 1

		Fold-change following infection	
**Mutation**	**Location**	**$293T_{rhm}$**	**$293T_{hu}$**
N656Y	CHR	34.2	4.5
N656Q	CHR	32.8	4.7
N656I	CHR	31.6	8.8
N656G	CHR	27.8	1.2
Q653L	CHR	26.8	2.8
N656R	CHR	24.2	3.3
Q653I	CHR	23.0	5.0
K588M	gp41	20.8	1.8
Q653N	CHR	20.6	3.0
Q653M	CHR	19.8	2.3
N554D	NHR	17.3	4.4
N656E	CHR	17.2	3.4
Q652F	CHR	15.6	3.0
Q653E	CHR	15.6	1.8
N656H	CHR	15.5	3.7
S649F	CHR	15.0	6.1
K588F	gp41	14.0	1.9
K655F	CHR	13.9	2.0
Q653F	CHR	13.5	1.1
N656S	CHR	11.4	3.1
Q652D	CHR	11.2	2.2
Q653Y	CHR	11.1	1.0
N656D	CHR	10.8	6.3
K655W	CHR	10.7	1.6
N656M	CHR	10.5	5.2
K655Y	CHR	10.1	1.9
R273K	gp120	4.0	10.6
E482K	gp120	7.9	11.7

Table S1. Fold-change relative to the wild-type residue for all mutants that were enriched greater than 10-fold following either $293T_{rhm}$ or $293T_{hu}$ infection in the DMS.
